# A review of machine learning experiments in equity investment decision-making: why most published research findings do not live up to their promise in real life

**DOI:** 10.1007/s41060-021-00245-5

**Published:** 2021-04-05

**Authors:** Wojtek Buczynski, Fabio Cuzzolin, Barbara Sahakian

**Affiliations:** 1 University of Cambridge, Cambridge, UK; 2 Fidelity International, London, UK; 3 Oxford Brookes University, Oxford, UK

**Keywords:** Artificial Intelligence, Backtest overfit, Investment management, Investment decision-making, Machine Learning, Investments, Investing

## Abstract

The numerical nature of financial markets makes market forecasting and portfolio construction a good use case for machine learning (ML), a branch of artificial intelligence (AI). Over the past two decades, a number of academics worldwide (mostly from the field of computer science) produced a sizeable body of experimental research. Many publications claim highly accurate forecasts or highly profitable investment strategies. At the same time, the picture of real-world AI-driven investments is ambiguous and conspicuously lacking in high-profile success cases (while it is not lacking in high-profile failures). We conducted a literature review of 27 academic experiments spanning over two decades and contrasted them with real-life examples of machine learning-driven funds to try to explain this apparent contradiction. The specific contributions our article will make are as follows: (1) A comprehensive, thematic review (quantitative and qualitative) of multiple academic experiments from the investment management perspective. (2) A critical evaluation of running multiple versions of the same models in parallel and disclosing the best-performing ones only (“cherry-picking”). (3) Recommendations on how to approach future experiments so that their outcomes are unambiguously measurable and useful for the investment industry. (4) An in-depth comparison of real-life cases of ML-driven funds versus academic experiments. We will discuss whether present-day ML algorithms could make feasible and profitable investments in the equity markets.

## Introduction

This article will analyze 27 peer-reviewed articles describing experiments in AI market forecasting over the past two decades (the details of the inclusion criteria are in Appendix A). Most of them focus on forecasting an entire market (proxied by a benchmark equity index). Virtually all of them claim great forecasting accuracy using one or more of the popular metrics such as mean absolute percentage error (MAPE), root mean square deviation/error (RMSD/RMSE), mean squared deviation/error (MSD/MSE), or mean absolute error (MAE). Many of them also employ a simple measure called “hit rate”, which measures the directional accuracy of a forecast. We will approach them from the perspective of their feasibility and applicability in real-world investment management.

In parallel, we will analyze the existing market data on ML-driven investment vehicles (“AI funds”). This data is limited in part due to understandable IP protection on the part of individual investment managers, but primarily due to the fact that based on available industry data and insights, the number of AI funds and their assets under management (AUM) are extremely low compared to the size of the industry.

The primary question we will try to answer is: could present-day AI be a consistently profitable investor in the real world? (please note: “consistent” does not mean “making a profit every day”; it means “making a satisfactory overall profit over a couple of years”). We will look at it from 2 perspectives: empirical and academic. We will see whether fund managers and academics have reached the same conclusions. Whether they had or had not, we will analyze why.

We grouped the articles from the perspective of their investment focus, breaking them into four primary categories:

- Market forecast—articles where ML algorithms attempted to predict the performance of one or more selected markets, proxied by a benchmark index. In this setup the focus is forecast and the ability to generate trading signals (buy, sell, hold, short). There is no active portfolio construction—benchmark index is the portfolio, and the algorithm does not make active allocation decisions.
- Individual equity forecast—articles where ML algorithms attempted to predict the performance of one or more individual equities. In essence, equity forecasting and market forecasting are very similar and fall into a broader category of time series forecasts.
- Bespoke portfolio construction—articles where AI algorithms attempted to predict the performance of a number of equities and build a profitable portfolio, autonomously determining asset allocations (weights).
- Other—all the research which does not fit into either of the categories above.

In this remainder of this section we will introduce a number of considerations and clarifications relevant to the use of AI in investment decision-making in general and to our article in particular.

### The definitional ambiguity of “AI fund”

In order to relate academic experiments to market practice (which we do in the Discussion section), our case studies focus on funds where machine learning is used (to a substantial extent) in the investment decision-making process. Please note that we will exclude funds where AI is one of the available tools for investment decision-making (e.g., MAN AHL) and focus on the ones where AI is the primary investment decision-maker.

### Investing versus trading

Trading is an act of buying or selling a financial instrument (equities, bonds, commodities—anything). It is execution of a buy/sell decision. In common parlance, trading is generally synonymous with speculation, i.e., short-term (or very short-term) transacting with a view to make instant profit—in extreme cases without any particular long-term strategy or sector/country/asset class focus. It is the latter meaning that gives trading most of its ambivalent (if not downright bad) reputation.

Investing means longer-term commitment that follows some sort of strategy, which has been stated at inception and accepted by the investors.

### Trading costs/“paper profit” versus real-world profit

Trading costs are charged by middlemen (usually brokers) for executing the transaction. Brokers connect multiple market players and find a “buy” for each “sell” and vice versa. Brokers charge a commission for their services.

Trading costs are a critical consideration. They are ever-decreasing, but they are still not negligible (especially for retail investors). If profit on a transaction is less (or equal) than the transaction cost, then the portfolio will make a loss (at best: break even). This logic will apply to each and every transaction and may make a difference between a simulated profit (even an impressive one) and a real-world loss.

### Short constraints

Most of the algorithms work with an implicit or explicit constraint of going long only (meaning that any individual asset’s weight w would be 100% >  = w > 0%). That limits profit opportunities, because the investor profits only when the value of their portfolio (be it entire market or a bespoke portfolio) goes up. However, investors may have both positive and negative views regarding the outlook of their investments,^1^ and are not restricted to profiting only from the positive ones. By going short, the investors profit from the value of their investments decreasing. There is an open question and discussion^2^ about the ethics and morals of shorting, but bottom line is that it is legal, simple, and common.

There are 2 likely reasons why most of the authors chose the long-only approach:

- It is much more intuitive.
- It has lower computational requirements than a long/short approach.

Additionally, it prevents the algorithm from recommending extreme weights on the long and short sides (given that portfolio weights *always* add up to 100%, an unconstrained long/short algorithm could recommend weights like − 999,900% and + 1,000,000%, which would not be feasible in the real world). Then again, this could be solved very easily by capping portfolio weights at, for example 100% >  = n >  = -100%.

In market portfolio (i.e., a benchmark index), all assets have positive weights, so the investor profits only when the market goes up.

### Laws and regulations

While there is no dedicated regulation covering AI in financial services (yet), there are existing regulations which can be applied to it (directly or indirectly).

Regulations vary by region and are not always equivalent in scope between different jurisdictions (typically the most developed markets will have the most comprehensive and forward-looking regulations). We will use 2 well-known pieces of regulation applicable in the UK (the first one being UK-specific and the second one being EU-wide).

From Dec-2019 onwards most financial institutions in the UK have been covered by the new and enhanced Senior Managers & Certification Regime (SMCR) [1]. The premise of SMCR is to explicitly name key decision-makers in the financial organizations and hold them personally (as well as legally and financially) accountable for their actions and decisions. Consequently, there would have to be a “human in the loop” for investment decisions made by a machine learning system. Even if the decisions were made solely by an algorithm, the named individual (portfolio manager) would still need to ratify them and by doing so assume responsibility and accountability under SMCR.

There are two tiers of conduct rules under SMCR, and a number of them can be applied to oversight of an investment decision-making AI (or lack thereof):

- 1^st^ Tier, rule #2: “You must act with due care, skill and diligence” (allowing an AI to operate unchecked and opaque is a likely breach of all three).
- 1^st^ Tier, rule #4: “You must pay due regard to the interests of customers and treat them fairly” (trusting a “black box” algorithm with clients’ assets is likely *not* paying due regards to their interests).
- 2^nd^ Tier, rule #1: “You must take reasonable steps to ensure that the business of the firm for which you are responsible is controlled effectively”

While SMCR focuses on conduct, EU-wide MIFID II regulation [2] covers a much broader area, including, crucially, suitability of recommended investment(s). Article 25(2) states very clearly: “When providing investment advice or portfolio management the investment firm shall obtain the necessary information regarding the client’s or potential client’s knowledge and experience in the investment field relevant to the specific type of product or service, that person’s financial situation including his ability to bear losses, and his investment objectives including his risk tolerance so as to enable the investment firm to recommend to the client or potential client the investment services and financial instruments that are suitable for him and, in particular, are in accordance with his risk tolerance and ability to bear losses.”.

An investor who put their assets in an AI-managed vehicle, which subsequently suffered substantial losses, may have a valid mis-selling claim against their asset manager on the grounds of suitability. Even if the “black box” nature of the algorithm was disclosed, then the client may still claim that the investment was unsuitable, because it might be altogether impossible to ascertain suitability and appropriate target market for an opaque strategy.

MIFID II actually goes a step further. One of its Regulatory Technical Standards (RTS; in this case RTS 6) [3] is wholly dedicated to requirements of investment firms engaged in algorithmic trading. One of the annexes covers disclosure requirements for investment decision-maker within a firm—which can be human (referred to by the EU lawmakers as “natural persons”) or an algorithm. MIFID II is likely the most cutting-edge and prescient piece of regulation touching on investment decision-making by an algorithm (which, despite there being some discussion as to what exactly constitutes an algorithm, would very likely cover AI).

Interestingly, even pre-MIFID II, there were precedents of clients suing their investment managers for disappointing investment performance.^3^ In absence of explicit regulation, the claim was negligence.

Cutting-edge thinking captured in MIFID II touches (indirectly) on a much broader consideration: legal status of algorithms. For now, it remains a purely theoretical discussion. Algorithms do not and cannot have legal personhood the way other non-human entities can (states, corporations). Consequently, for *any* investment decision made by an algorithm, liability will be with an individual who approved the decision (in SMCR and similar frameworks), an investment company, or both.

## Discussion

### Thematic review

Our analysis is dominated by whole market prediction [4–18] with a number of instances of individual equity forecasts [19–27]. The two have so much in common that analytically they belong in the same category (price series forecasting), even though from investment perspective they do not (allocation into specific market(s) is very different from individual stock picking). There are 2 cases of bespoke portfolio construction [28, 29] and one case which genuinely falls into “Other” category [30].

Among experiments attempting index forecasting, 9 [4–8, 12–15] attempted to predict the exact value of the index in the future, while 6 [9–11, 16–18] attempted to predict the directional change only (i.e., whether the index would go up or down in the future). We favored the latter approach because it lends itself to very clear, unambiguous statistics (% directional accuracy during the training period aka “hit rate”).

Regrettably, *none* of the individual equity forecasting experiments used hit rate as measure of its predictive accuracy. This group of experiments was characterized with the most diverse measures, sometimes not measuring predictive accuracy/error at all, and focusing on portfolio return. Return is almost as unambiguous a metric as hit rate, it is not entirely comparable across markets and periods in time though. The lack of uniformity across forecasting accuracy measures is something we cannot easily explain—we would expect seeing the same metrics we saw in market forecasting (hit rate, MAPE, MAE etc.).

Out of 27 experiments, 9 employed ensembles—6 explicitly [12–14, 20, 21, 24] and 3 implicitly [15, 27, 30] (i.e., they do not call their setups ensembles, but the model characteristics seem to match the characteristics of ensemble).

In our literature analysis, we identified 2 distinctive types of ensembles. We can call the first type “single-stage ensemble”, whereby the constituent models work together to deliver the prediction(s). We can call the second type “multi-stage ensemble”, where different models are used at specific stages of prediction (e.g., one will be used for input selection and other one(s) for prediction employing those inputs, or one will be used to optimize the structure of the other). “Single-stage ensemble” and “multi-stage ensemble” are not industry standard terms—we came up with them over the course of writing this article, but we believe that increasing specialization and differentiation of ensembles warrants introduction of new terminology.

### Representativeness of results/backtest overfitting

The concept of “backtest overfitting” was introduced to the investment realm by acclaimed mathematician David Bailey et al. in 2014 [31]. Bailey writes “Overfitting is a concept borrowed from machine learning and denotes the situation when a model targets particular observations rather than a general structure.”. The contrast between academic results and the industry outcomes led us to question representativeness of results presented in reviewed articles. We were also pointed in that direction by a number of authors candidly admitting that the results they presented are either the best ones out of a larger set, or an average of multiple experiments ran in parallel. We revisited all the experiments. For each of them we wanted to know how many configurations of the same model/test runs/parallel simulations were run in the *testing* phase (we are not concerned about multiple setups being used in training phase—that is exactly what training phase is for).

A number of researchers were candid and explicit in using more than one model set-up in testing phase. Conversely, no author stated explicitly and unambiguously that they used *only one configuration* in testing (a number of researchers implied it). A number of researchers implied it. We realized that in this sensitive area there is considerable potential for errors which would cause the authors understandable upset. Consequently, we proceeded on the following bases:

- Unless the author(s) explicitly stated otherwise, we would by default assume that they used just *one* model configuration.
- We note that 9 experiments (out of 27) were assumed to have used just *one* model configuration.
- We disregarded 2 further experiments in which we found the authors too vague to make any inferences regarding the number of configurations, and 1 more in which there had been multiple configurations used, but the exact number was not provided.
- We analyzed the remaining 15 experiments more closely. In order to minimize the potential for an upsetting misunderstanding, we look at the 15 on anonymized basis.

The average number of model configurations used in the 15 experiments was 70.7. That immediately disqualifies them from real-life investment perspective. However, the median was a more reasonable 5, which means that the mean was skewed by a number of outliers (which indeed was the case). Still, 5 is no better from real-life investment perspective. The only number which works in real-life investing is 1. We referred to the practice of picking the best-performing model as “cherry-picking”. It is obviously very closely related to Bailey’s backtest overfitting. The latter refers to the process of matching one out of a number of outcomes to training data, while the former refers to presenting it (implicitly or explicitly) as a representative result.

Separately, we also note that multiple experiments tested the robustness of their results (e.g., through significance testing). However, this was always done on a “cherry-picked” set of results, which invalidates the whole concept. With a sufficiently large pool of results, one will eventually match the observable market data (even if it’s random). Same can be applied to significance testing.

That means that the practical value of (at least) 15 articles is, unfortunately, zero—and that is taking a very cautious approach. We believe the number of articles which employed more than one model configuration in testing phase may be much higher than 15.

### The use of averaged forecast errors as evidence of algorithm’s forecasting accuracy

Within the set of articles we analyzed, 12 [4, 6–8, 12–14, 20, 25, 27, 29, 30] used one or more mean forecast errors such as MAPE (mean absolute percent error), RMSD/RMSE (root mean square deviation/root mean square error), MSD/MSE (mean squared deviation/mean squared error), and MAE (mean absolute error).

9 experiments [4–6, 9–12, 17, 18] used hit rates (the % of cases when the *direction* of the market was forecast correctly, but not the exact value).

In total 18 out of 27 experiments used one of the abovementioned standardized forecasting accuracy metrics.

#### Hit rate

Hit rate can be objectively compared across different experiments. Hit rate is simple, unambiguous—and more reductive than all other measures. Hit rate only indicates whether *direction* of the forecast matched the direction of the underlying instrument’s price change—it does not measure the degree of such change. However, knowing the direction of instrument’s price change can be just as profitable and useful as knowing the exact magnitude of such change. We will simply invest in the assets whose price is forecast to go up over the timeframe of our interest (which can vary from daily to yearly). If our strategy permits shorting (i.e., betting on a value of a security to fall and profiting from that fall), we can at the same time short the assets forecast to decrease in value, and profit from that as well. There is certain disadvantage with using hit rates only, which stems from having less information than exact price forecast. We may invest in an instrument whose price will indeed go up over selected timeframe—but the price appreciation may be marginal. If we forecast Vodafone, HSBC, and Whitbread all to go up over the course of one year and invest in them in equal proportions, and their annual price increase is 7%, 0.5%, and 13% respectively, it is likely that we will in hindsight consider HSBC to be a suboptimal investment. Having a forecast of actual % price changes would help us allocate much more efficiently and profitably.

In short, we consider hit rate a great metric for comparisons across multiple experiments, but insufficient from the perspective of a serious investor.

**Fig. 1:**
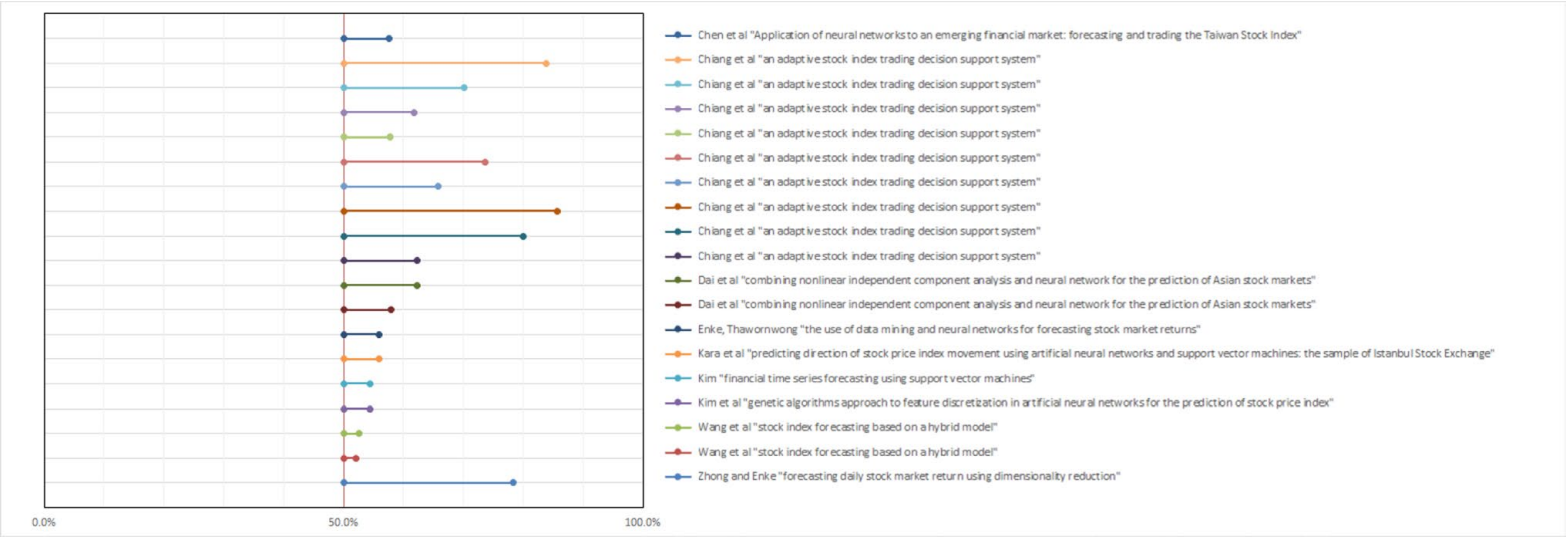
Forest plot of all applicable experiments’ hit rates

Interestingly, the nine experiments whose authors disclosed hit rates were among the ones whose performance metrics were very mixed. Half of those had performance only slightly above random guess for a binary classification problem [Enke and Thawornwong 47%–69% depending on the algorithm; Chiang 50%–62% depending on the market; Kim 52%–58% depending on algorithm selected; Kim 61.7%; Zhong 56%–58% depending on the algorithm]. The other half had much higher results [Dai 72%–86% depending on algorithm and market; Chen 73%–83% depending on prediction horizon; Wang 70%–84% depending on the market; Kara 71%–76%]. That is another indication that present-day ML algorithms may not be quite ready to make investment decisions on a level more accurate and more profitable than humans (Fig. 1).

#### Mean error measures

Mean Absolute Percent Error (MAPE)

MAPE is a very popular measure, used in 9 articles. Its apparent strength is that it is expressed in % and thus seemingly comparable and standardized. MAPE is an average of absolute % differences between the forecast value and the actual value.

1 $$ {\text{MAPE}} = \frac{100\% }{n} \mathop \sum \limits_{t = 1}^{n} \left| {\frac{{A_{t} - F_{t} }}{{A_{t} }}} \right| $$

Formula 1: MAPE.

Root Mean Square Deviation (RMSD)/Root Mean Square Error (RMSE)

The second most popular unit of forecasting accuracy was root mean square deviation (RMSD), otherwise known as root mean square error (RMSE), which was used in 6 articles.

2 $$ {\text{RMSD}} = \sqrt {\frac{{\mathop \sum \nolimits_{t = 1}^{n} (F_{t} - A_{t} )^{2} }}{n}} $$

Formula 2:RMSD/RMSE.

We consider RMSD/RMSE suboptimal for the purpose of measuring forecasting accuracy of financial time series. Unlike MAPE or hit rate, it does not have intuitive, standardized units. In case of index forecasting RMSD/RMSE will be expressed in points, because this is the unit of index value (consequently, talking about “index price” is not strictly accurate, but it is a widely accepted figure of speech). In case of individual equities forecasting RMSD/RMSE will be expressed in monetary amounts. Furthermore, RMSD/RMSE is not easily comparable, because it is a function of absolute values (both forecast and actual). That makes RMSD/RMSE non-comparable not only across different experiments, but also across different time series. Furthermore, RMSD/RMSE is very sensitive to outliers (due to squaring), even if they are infrequent. In the fourth simulation of our experiment we had a total of 7 outliers (substantial differences between forecast FTSE 100 value and its actual value) out of 253 data points. Those 7 outliers raised RMSD/RMSE from 368 (simulations #1, #2, and #3) to 497. More fundamentally, we see no value and no justification for using RMSD/RMSE as a measure of forecasting accuracy in financial time series: not only is it not comparable, not scalable, and sensitive to outliers, but it is not telling us anything meaningful even when we are looking at just one time series on its own (i.e., when considerations of comparability and scalability do not apply).

Mean Squared Deviation (MSD)/Mean Squared Error (MSE)

2 articles used mean squared deviation (MSD), otherwise known as mean squared error (MSE)

3 $$ {\text{MSD}} = \frac{{\mathop \sum \nolimits_{t = 1}^{n} (F_{t} - A_{t} )^{2} }}{n} $$

Formula 3: MSD/MSE.

MSD/MSE are calculationally very closely related to RMSD/RMSE—MSD/MSE sum squared errors, but do not take square root of them (we could conceptually compare MSD/MSE to variance and RMSD/RMSE to standard deviation, which is a square root of variance). The criticisms applicable to RMSD/RMSE also apply to MSD/MSE: It is sensitive to outliers, does not have a standardized unit, and is not comparable across different time series (non-comparability is even more extreme for MSD/MSE than it is for RMSD/RMSE due to lack of taking the square root). We see no value in MSD/MSE as measures of forecasting accuracy.

Mean Absolute Error (MAE)

2 articles used Mean Absolute Error (MAE) as a measure of forecasting accuracy

4 $$ {\text{MAE}} = \frac{{\mathop \sum \nolimits_{t = 1}^{n} \left| {F_{t} } \right. - \left. {A_{t} } \right|}}{n} $$

Formula 4: MAE.

Just like MSD/MSE and RMSD/RMSE before, MAE has its shortcomings of non-uniform unit and non-comparability across different time series. Unlike MSD/MSE and RMSD/RMSE, MAE is less sensitive to outliers due to lack of squaring. It is also much more straightforward and more intuitive to understand. It may be a helpful metric in analysing a standalone time series. It could also be used in comparing forecasting accuracy of different models applied to the exact same time series.

#### Experiment—creating “on average profitable” time series

We consider mean forecast errors to be a flawed measure Our rationale is as follows: a couple of highly inaccurate forecasts may be all it takes to deplete the assets of a portfolio beyond the point of plausible recovery, or beyond the point of investors’ risk tolerance (at which point they will crystallize the loss and withdraw whatever assets they have left). On a long enough timeline (e.g. 1 year) such model may be on average accurate with its forecasts, and in the end the handful of severely inaccurate forecasts will be averaged out, and the mean forecast error might indicate a robust and successful model.

All of these metrics also disregard the fact that in investments the final outcome is a result of geometric compounding of all individual daily outcomes in sequence, and *not* an average.

We noted that experiments using mean forecast errors almost universally reported accuracy that bordered on phenomenal (90%–98%). In many cases these metrics were presented as conclusive proof of accuracy, robustness, and overall excellence of a given model.

Rather than question specific experiments and their results (where we do not have the complete set of data), we decided to put these measures to the test ourselves.

In our experiment, we took daily FTSE 100 equity index prices from 2018 (253 data points). We wanted to see whether we can produce time series of simulated (doctored) forecasts which would exhibit very low MAPE (we arbitrarily set it at 5%, implying model was 95% accurate), whilst at the same time being useless from the investment perspective. We used MAPE as our primary reference because of all the mean error measures, MAPE is the only one expressed in % and thus unambiguous.

We devised the following scenarios:

1. The first simulated forecast overestimated actual index price by 5% each day. MAPE was thus 5%. Directional accuracy was 100% (more on that later).
2. The second simulated forecast underestimated actual index price by 5% each day. MAPE was thus 5%. Directional accuracy was 100%.
3. The third forecast would sometimes overestimate the actual index price by 5%, and sometimes underestimate it by the same amount. MAPE was still 5%, directional accuracy was 47%.
4. In the fourth simulation, the forecast was usually within 5% range from the actual index price, except for a handful of extreme outliers (under- and overestimations by 30%). MAPE was still 5%, directional accuracy was 57%.

We are not in the position to be able to state as to which of the above scenarios would be most disadvantageous to a real-world portfolio, because we would need to make multiple assumptions about its structure, investment horizon, long/short constraints, risk tolerance etc. —it is not the point of our simulations. The point of our simulations was to show that MAPE is a very weak indicator of robustness and consistency of a time series forecasting model and we believe we accomplished that.

**Fig. 2:**
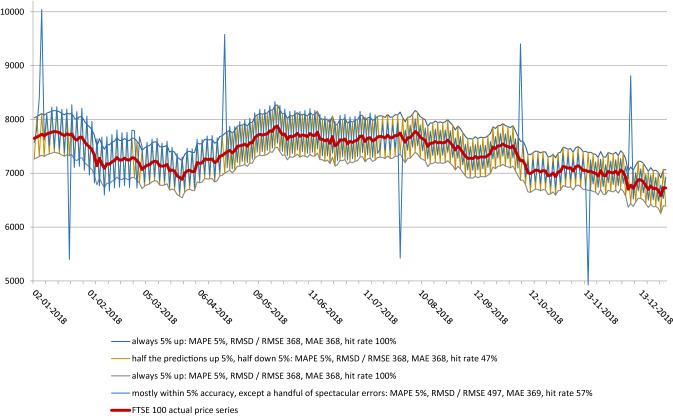
Simulated forecasts time series

Going back to the point about real-world applicability, the simulations where directional accuracy was in the region of 50% show empirically that the forecast (in the binary realm of UP or DOWN) was no better (or not much better) than a flip of a coin, which disqualifies it entirely. The 2 simulations whose directional accuracy was 100% were extremely unrealistic in order to help us make another point: no metric on its own gives a complete picture of a model’s robustness and forecasting accuracy. The hit rate of 100% does imply profitability in certain strategies (namely daily speculation), but a forecasting model which under- (or overstates) index price 100% of the time is unlikely to be relied on by investment professionals (Fig. 2).

Summary

In conclusion, we see shortcomings with all the abovementioned, mean error-based metrics. Hit rate is relatively most universal, clear, and comparable of all of them, but at the cost of reduced informational value. Regardless of the metric (MAPE, RMSD/RMSE, MSD/MSE, MAE) we consider “mean” to be the four-letter word of financial time series forecasting. When looking at outliers, the choice appears to be between bad and equally bad: metrics which are sensitive to them (RMSD/RMSE, MSD/MSE) are easy to “hijack” by extreme values; metrics which are less sensitive to them (MAPE, MAE) make it easier for huge (potentially devastating) forecasting errors to “average out”. Furthermore, all of these metrics (including hit rates) disregard temporal effect and compounding, which is critical in finance: one sufficiently large loss (especially towards the end of the investment period) could wipe out months or years’ of compounded (unrealized) profits—but on average the returns and the forecasting accuracy could be presented as very high.

Consequently, in our view the only truly meaningful measure of model’s forecasting quality is a complete time series of forecast prices, which can be compared against actual market prices. It will also allow a comprehensive independent analysis. Profit (even high profit) is insufficient because it will not give insight into model’s consistency over time and the variability of the forecasts vis-à-vis actual values.

### Academic results versus investment industry outcomes: a cognitive dissonance

Majority of articles in our review claimed good or great forecasting accuracy (particularly using mean errors—less so when using directional accuracy), oftentimes exceeding 90%, and sometimes exceeding 98%. This is truly phenomenal accuracy, which leads us to 2 logical conclusions:

1. If ML algorithms repeatedly and verifiably delivered forecasting accuracy in the range of 90%, we would expect them to proliferate in the investment management industry.
2. If ML algorithms repeatedly and verifiably delivered forecasting accuracy in the range of 90%, we would expect the few known deployments of ML-driven investment funds to deliver unparalleled returns.

Neither of the above is the case in the investment management industry.

Regarding proliferation of ML in investment management industry, it is not always easy to obtain unambiguous statistics. Investment management industry is huge and segmented among various very different types of entities (mutual funds, pension funds, ETF’s, hedge funds, sovereign wealth etc.), which are characterized by varying degrees of transparency and disclosure. According to alternative market research firm Preqin and Wired magazine, as of 2016 there were approx. 1,360 “quant” hedge funds, i.e., those for which majority of investment decisions was made by computer models. Of the entire investment world, hedge funds are most likely to deploy cutting-edge models: they are open to sophisticated investors only (which excludes private individuals, they have much lower disclosure and reporting requirements, and can pursue almost any investment strategy they please, as long as their investors accept it. According to Preqin, the aforementioned 1,360 hedge funds had combined assets under management (AUM) of USD 197bn [32]. USD 197bn is an enormous amount of money, but that will count *all* quantitative strategies, including rules-based ones which will not qualify as ML. While we can only speculate, it is likely that hedge funds utilizing fully-fledged AI’s in their decision-making process are likely a fraction of the 1,360/USD 197bn number. Focusing on AUM, USD 197bn pales in comparison to total AUM of the hedge fund industry (USD 3.05Tn [33]). Even that exorbitant amount is almost negligible in comparison to total AUM of 500 largest investment (non-hedge) fund managers, which at the end of 2017 stood at USD 93.8Tn [34]. Please note that USD 93.8Tn does not even reflect AUM of the entire investment management industry—it is just the 500 largest players globally.

Financial Services Board Nov-2017 report titled “Artificial intelligence and machine learning in financial services” [35] corroborates our conclusions by stating “[…] ‘pure’ AI and machine learning players have about USD 10bn in AUM, but this figure is growing rapidly”. The figure is based on FSB’s discussions with investor focused on this particular area, which makes it anecdotal, but we are confident that FSB—a major global financial body comprising regulators, central banks, and ministries of finance from dozens of jurisdictions—would not have published a figure it was not confident about. We appreciate that said AUM likely “grew rapidly” since Nov-2017, but we doubt it exceeded low hundreds of billions worldwide.

The FSB report highlights one more thing: the challenge of collating reliable data in this niche area. It proves that data (particularly AUM) is not readily available even to a global financial association.

It immediately becomes apparent that proportion of AI-managed AUM to broader industry AUM is somewhere between marginal and negligible. That directly (and empirically) contradicts conclusion #1 above. There may be a “middle ground” explanation that models and algorithms *do* deliver, but the industry adoption takes time.

That takes us to conclusion #2 (“If ML algorithms repeatedly and verifiably delivered forecasting accuracy in the range of 90%, we would expect the few known deployments of ML-driven investment funds to deliver unparalleled returns”). This conclusion is more anecdotal and proving or disproving it is largely dependent on access to industry data. As we discussed above, ML-driven funds are most likely to be hedge funds, which are much more secretive in their nature, and have far lesser disclosure requirements than regular mutual funds or ETFs. It is therefore theoretically possible that there indeed are some ML-driven hedge funds which make phenomenal profits. To extrapolate further, it is also possible that management have an interest in keeping this information as guarded as possible (e.g. to protect their intellectual property). This is possible, but based on our knowledge of the industry, unlikely for a couple of reasons:

- The kind of technological infrastructure required is likely to be expensive (whether it is on-premise or outsourced to the cloud), not to mention specialist staff. That means that established, brand-name funds (hedge funds and otherwise) are the natural candidates. It is unlikely that they would manage to keep performance of one or more funds a secret for very long—not because of breach of secrecy, but because it would be against their interests.
- The abovementioned interest is AUM growth. All funds who manage assets on fiduciary basis (i.e., other people’s money, not their own) make their money by charging a % of AUM. The rule is very simple: the greater AUM, the more the fund itself profits. It would therefore be very much in fund’s interest to broadcast their performance as vocally as possible, in order to attract more AUM.
- Most funds (perhaps with the exception of fully private structures) are covered by market data vendors who sell this data onto their clients. For larger entities it would be exceptionally difficult to evade such scrutiny, and it could be interpreted as sign of problems (up to and including fraud—the memory of Bernard Madoff’s audacious decades-long scam will not fade anytime soon).

Some of the high-profile news stories in the ML-driven investment space have been those of underperformance and/or liquidation. Aidya was a Hong Kong-based ML-driven hedge fund employing ensemble models. It was created and run by AI legend Ben Goertzel. Aidya delivered 12% on its first day—and liquidated after less than a year. One of us (WB) had the chance to speak to Goertzel in person, who confirmed that fund was closed due to disappointing performance. Goertzel is not currently active in the ML-driven investment space.

Sentient Technologies, a high-profile start-up hedge fund which attracted USD 143 m in VC funding for its evolutionary algorithms-based trading strategies, liquidated in 2018. It is not entirely clear whether Sentient fulfilled our criteria of an investment vehicle, or whether it focused on short-term speculation. In any case, it was formed as a fund, and perceived by industry as such. The fund made a modest 4% return in 2017 and none in 2018, when it was liquidated.

Rogers AI Global Macro ETF was launched in June 2018. It employed AI in investment decision-making capacity. It operated for just over 1 year (from June 2018 to July 2019) and during that time made close to no profit (its opening price was USD 24.97, and its closing price was also USD 24.97. The fund paid approx. 0.60 USD in dividends).

EquBot’s AI Equity ETF (AIEQ) is another high-profile non-success story. Powered by IBM’s Watson, it lost 7.28% in 2018 (its benchmark, S&P 500 lost 4.75%) and earned 31.14% in 2019 (S&P earned 33.07%). Its modest underperformance and continued existence are the closest we have come across to an AI fund success story to date.

**Fig. 3:**
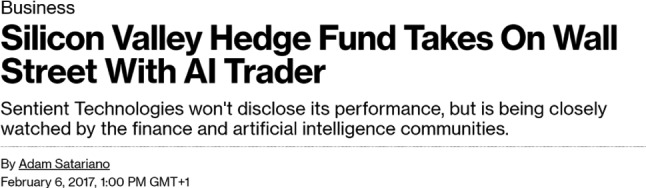
Bloomberg article headline from 06-Feb-2017 [36]. Source: Bloomberg Finance L. P

**Fig. 4:**
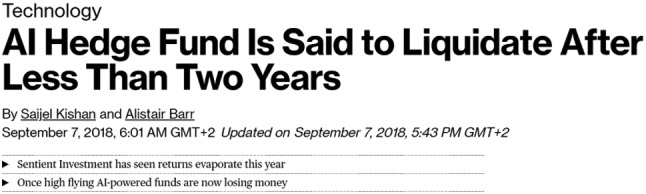
Bloomberg article headline from 07-Sep-2018 [37]. Source: Bloomberg Finance L. P

We note that due to nature of investment industry where consistently underperforming funds tend to be shut (especially those with short track record, limited pools of capital, and generally speculative in nature—all of which apply to AI funds) against the backdrop of AI hype and likely high expectations, the distance between boom and bust tends to be exceptionally short (Figs. 3, 4). The short time it takes for yesterday’s rising star to shut down and liquidate exacerbates the contradictory messages in the media, as exemplified by the 2 Bloomberg headlines referring to Sentient Technologies.

So much for high-profile anecdotal cases. Aggregate-level picture is an intriguing counterpoint. Niche index vendor Eurekahedge compiles its AI Hedge Fund index, which is often referenced in industry articles. The company makes it clear that the index tracks “hedge fund managers who utilize artificial intelligence and machine learning theory in their trading processes” (https://www.eurekahedge.com/Indices/IndexView/Eurekahedge/683/Eurekahedge_AI_Hedge_fund_Index), so it is not an index of funds which invest in AI-related companies, but those which employ ML in their investment decision process. The index is base-weighted as of Dec-2010. The index currently has only 13 constituents, which makes it highly sensitive to outliers. Furthermore, each of the 13 funds is equally weighted in the index, which is not a standard index methodology. Standard methodology would weigh the funds by the size of their AUM (i.e., larger funds would have a correspondingly higher weighting in the index than the smaller ones), although it is possible that AUM disparities in such a small and niche universe of funds are so large that weighting by AUM would lead to one or two funds completely dominating the index.

**Fig. 5:**
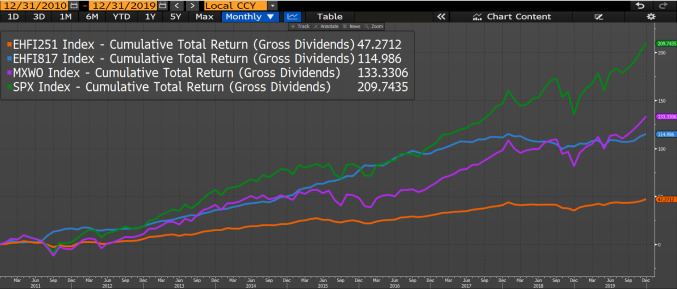
Cumulative performance of Eurekahedge AI (EHFI817 Index), Eurekahedge hedge fund (EHFI251 Index), S&P 500 (SPX Index) and MSCI World (MXWO Index) indices. Source: Bloomberg Finance L. P

These considerations aside, let us have a look at the returns. From Jan-2011 to Jan-2020 Eurekahedge AI (EHFI817 Index in the Bloomberg screenshot below) has substantially underperformed 2 global benchmark indices, S&P 500 (SPX Index) and MSCI World (MXWO Index), with cumulative returns of 114.98%, 209.74%, and 133.33% respectively. However, there is no specific reason for us to compare Eurekahedge AI to those 2 particular benchmarks—they are just very well known, and are very popular points of reference (“this is how much I would have made had I simply invested in the market”). It is exceptionally difficult to find reference benchmarks to compare Eurekahedge AI to. Broader hedge fund index (EHFI251 Index) from the same Eurekahedge family delivered a total return of 47.27% (Fig. 5).

Transparency of market data and the methodologies of its aggregation tend to decrease as we move into increasingly niche areas (which hedge funds, and even more so AI hedge funds, are). This does not mean that vendors’ data is not trustworthy. It means that it cannot be independently verified and recalculated (which is the case with all alternative and/or illiquid assets) the way, for example, S&P 500 can be. It also means that performance figures may differ, even within seemingly similar or identical asset classes—this will be based on the inclusion criteria, the number of assets within the index, index construction methodology etc.

As a counterpoint to Eurekahedge data we also have some data from Preqin, which also tracks performance of hedge funds in general, as well as AI hedge funds. Preqin defines its universe similarly to Eurekahedge (“hedge funds that use AI to help with trading”). It includes far more funds though: 152 versus Eurekahedge’s 13 (we suspect that Preqin may be more “inclusive” than EH), and the results differ too: Preqin’s AI hedge fund universe generated 26.96% return in 3 years from Aug-2016 to Aug-2019, and its all hedge fund benchmark earned 23.87% [38]. Eurekahedge data for analogous period indicates returns of 7.78% for its AI hedge fund index and 12.63% for its all hedge fund index.

26.96% versus 7.78%, and 23.87% versus 12.63%—those numbers are clearly very different. They do not disqualify either of the vendors, they just indicate that there are substantial differences in methodologies and fund selection (inclusion) criteria. Also, most importantly and conclusively, both sets of returns pale in comparison with S&P 500 (45.63% total return over the same period) and MSCI World (37.01%). Regardless of the vendor, the case for AI hedge funds’ underperformance seems to be corroborated rather than disproven. The only disagreement can be over the scale of their underperformance.

In short: there is no conclusive evidence of *any* ML-driven investment funds delivering spectacular returns at scale. All market data indicates substantial underperformance compared to benchmark indices.

## Conclusions

### Explainability and transparency

All the experiments in our review were at their core “black boxes”. This negatively contrasts with human-made decisions, in which the portfolio manager does usually have a solid rationale and basis for each investment decision.

Lack of explanation on how an algorithm arrived at a particular forecast or recommendation is suboptimal in the experimental (theoretical) context, but very risky (if not unacceptable) in practical context, where there would be real investors’ money at stake. It is also likely to raise concerns of regulatory and/or legal nature.

### Accuracy and feasibility of AI market forecasts/“cherry-picking”

We noted multiple times that most of the experiments were not realistic and/or feasible in real-world investment management practice. That was mostly due to poor performance measurement and running multiple versions of the model in parallel. A number of authors disregarded trading costs, and even fewer tried any sort of investment simulation as a means of testing their algorithms’ performance.

We cannot conclusively state whether the algorithms tested in our review are or are not successful in forecasting of financial time series because we do not have complete underlying data for each experiment. However, through inference, qualitative, as well as quantitative analysis our findings suggest that most—if not all—AI models likely perform more poorly than claimed. This is driven by 2 factors:

- The use of inadequate performance metrics.
- Results selection (“cherry-picking”).

A number of articles were candid in disclosing that multiple configurations of the “core” algorithm were used (in extreme cases up to hundreds). In almost all the cases the authors presented their highest-performing model as the primary product of their experiment. This is completely incompatible with real-world investment management. Running multiple variants of the same strategy in parallel, and then presenting the most successful one as representative would be misleading and very likely illegal (misrepresentation of fund performance).

Just one version of an algorithm should be run in testing (we have no problem with trying out multiple configurations in training or calibration stages, as those represent “learning” part of the process). This would be representative of a real-world investment setup and therefore realistic.

A discussion of performance outliers was lacking in almost all the articles. In different disciplines outliers can be justifiably disregarded as insignificant to the bigger picture, random errors, calibration errors, etc. Financial time series are different in that the investment outcome is a result of the entire, chain-linked, compounded time series. Most of the authors appeared to discount temporal effects entirely.

Our conclusion is that authors—overwhelmingly hailing from computer science background—seem to approach financial time series without paying due attention to their unique characteristics. In our view this is the key reason why there is such a huge divergence between academic literature (and its claims) and limited (and not always successful) adoption of AI in the investment decision-making process in investment management industry.

### Performance measurement

We found most methods of measuring performance accuracy unfit for purpose in the context of financial time series forecasting (especially those based on average forecast error) —and that is the key finding of our analysis. We conclude that only full disclosure of forecast time series (which will reveal divergence from actual equity index time series, all the outliers, dispersion of forecast values etc.) would be a way to evaluate robustness of an AI algorithm vis-à-vis human analyst.

### Improvements over time (longitudinal analysis)

The timeframe of our analysis was close to 20 years. We approached our review expecting substantial improvements in forecasting accuracy and sophistication of models over time. We were correct about the latter: models did become more complex over time, with an observable shift to ensembles of different kinds.

We were expecting models to perform at a certain initial level (we had no specific expectations as to what that level would be) back in 2000, only to see their performance markedly improve between 2000 and 2019.

On the basis of forecasting errors (MAPE etc.), researchers claimed very accurate forecasts since the early 2000′s ((Enke, et al., 2005) claimed RMSE below 2% in their 2005 article, setting the bar very high for subsequent experiments).

On the basis of hit rates we observed no improvements in forecasting accuracy over time (in fact (Chiang, et al., 2016) and (Zhong, et al., 2017) had lower hit rates than (Kim, et al., 2000) or (Chen, et al., 2003)).

If performance improvements were reflective of advances in the field of AI and growth in available computing power, those advances should be substantial. We observed nothing of that kind.

### Legal and regulatory considerations

Over a decade after the financial crisis, the finance industry has implemented a number of laws which are applicable (directly or by extension) to algorithmic investment decision-making. By contrast, we note that *none* of the 27 papers we reviewed gave any thought to legal or regulatory considerations.

### Market forecasting versus bespoke portfolio construction

We approached our review without any specific expectations regarding the distribution of scholarly coverage among topics of our interest (whole market prediction/bespoke portfolio construction/other approaches). It quickly became apparent that market forecast is the most popular research area (15 out of 27 articles). It is followed by forecasts of individual equities (5 articles), which is very similar to index forecast. Another 5 experiments cover multiple equities forecasting within a simplified portfolio structure, and only 2 attempt bespoke portfolio construction. Upon reflection, we begin to understand why. Portfolio construction is a multi-stage process consisting of a number of distinct, specialized tasks. Expecting AI to do most or all of them (and to do them well) is unrealistic and probably not very practical (not to mention regulatory considerations or likely investors’ concerns regarding no human in the loop). It makes much more sense to employ AI in one, specialized task towards which it is better-equipped (time series analysis and forecast).

We were surprised to find no examples of AI optimizing portfolio allocations (within a finite set of stocks to choose from) to get optimal risk/return trade-off, given that optimization is AI’s specialism.

### The potential limitations of Machine Learning algorithms

We found one very notable absence in each of the 27 experiments we analyzed—none of them explicitly addressed forecasting extreme market events (for the avoidance of doubt: in finance, “extreme” means “extremely adverse” —no one has ever complained about extremely high profits or too high returns). A number of articles did include periods of markets downturn in their forecasts (especially around 2008), but we believe their authors may have missed an important thing: the Machine Learning algorithms seem to be “organically” constrained by historical data they were trained on.

We have no way of knowing whether researchers who conducted 27 experiments in our review considered extreme events and whether their algorithms were robust enough to forecast events so extreme that they were not captured in their training sets. We only have to look back to the initial wave of COVID-19 in early 2020 as an example: on 12-Mar-2020 FTSE 100 index fell 10.87%—its highest loss since 1987 and its third-worst day performance in the entire index history (dating back to 1984). 16-Mar-2020 was also the second-worst day for Dow Jones Industrial Average (-12.93%) in its 124-year long history, worse than the worst days during the 1929 crash. The spring of 2020 also brought some of the most spectacular rebounds in equity indices history: on 24-Mar-2020 FTSE 100 had its second-best (+ 9.05%) and DJIA had its fourth-best day ever (+ 11.37%).

The open question is whether any of the Machine Learning algorithms in our study could even theoretically forecast gains and losses higher than anything contained in their training set. The more fundamental open question is whether authors considered such scenarios when designing their algorithms.

### Ethics

Most of the experiments in our review focused on forecasting broad market indices. This immediately precludes shorting (as standard market indices are all long-only). The investor may choose to short a market based on a negative forecast, but this is an ethical choice of an investor, not of the ML algorithm. By similar logic, forecasting an index of hundred(s) or even thousands of companies treated as a whole is a reflection of the investor’s ethics, not the algorithm’s. Many indices will include companies that are morally objectionable to some investors (for example fossil fuel or defence companies), but the algorithm is not given a choice. Consequently, we consider all the experiments in which AI was forecasting an equity index as “ethically neutral” (as mentioned in the Discussion section, vast majority of the experiments fell into that category). The experiments that forecast individual equities selected a priori by the researchers also fall into that category.

The only experiments in which “active” ethics considerations applied were the ones in bespoke portfolio construction. We note that the authors made no mention of any ethics guidelines for their algorithms to follow or learn.

In summary, we note that none of the authors explicitly addressed ethics (even as a theoretical consideration). It was a non-consideration across the entire study.

## Future recommendations/best practices

### Explainability and transparency

“Black box” forecasts and investment decisions violate existing investment regulations on the grounds of knowledge of products offered as well as suitability. These regulations were put in place to protect the investors. Most of the algorithms we reviewed appeared to be “black boxes by design” (especially the ones based on neural networks) and thus nearly impossible to be made explainable. We do not know how easy (from the engineering perspective) it will be to make algorithms explainable, but we are confident that the requirements for explainability and transparency will only increase. We are also confident that regulators, investors, and investment management firms will not accept black box tools to make investment decisions. We see the need for a fundamental change in the way financial time-series forecasting is approached by academia. This change is also essential in making academic research relevant and applicable to real-world investment practice.

### Clear protocol for performance measurement

We noted that at least 15 out of 27 articles ran multiple configurations of their algorithms in parallel, either averaging or picking the best results (“cherry-picking”). Averaging is an honest reflection of performance from the statistical perspective, but it is not compatible with real-world investment practice. Investment managers do not create multiple “clones” of the same original portfolio, trying multiple (potentially contradictory) strategies in parallel and averaging the returns.

We have no reservations against trying multiple configurations in the training phase. However, in testing phase there should be *only one* model configuration. Its performance should be disclosed in full—not in the form of a mean prediction error, but as a complete forecast time series.

Our analysis has conclusively proven the shortcomings of all popular means-based metrics for forecasting accuracy. We believe that devising entirely new metrics or ratios could be an interesting challenge for researchers in the field of quantitative finance. Our belief is that two metrics may be required to quantify the accuracy of a forecast:

- One for tracking overall fidelity/accuracy of the forecast (conceptually similar to ex-post tracking error).
- One for capturing and “penalizing” large individual outliers.

The 2 metrics proposed above could be considered as equivalents of precision and recall in Machine Learning classification. For the avoidance of doubt, those metrics would exist in addition to (and not instead of) complete time series forecast. They could be used for standardized comparisons of multiple forecasts, but they would not 100% conclusive on their own. Their added value would be supplemental.

### Accountability and liability

Legal and regulatory considerations may be overlooked in academic experiments focused on technology, but they come to the fore in real-world applications.

Even though the investment decision-maker may—even in light of present-day regulations—be an algorithm, there will be human person(s) who authorized its deployment. Given that an algorithm cannot have legal status/personhood under present-day laws, the liability will be with the investment company (as a legal entity) and, increasingly, the management, who may have personal financial liability.

It could be argued that technological aspects of ML could be “decoupled” from legal and regulatory considerations—the former should be tackled by engineers and the latter by compliance specialists and lawyers. Our industry experience and analysis of regulatory guidances worldwide are contrary to that: there is a growing trend for assembling interdisciplinary AI teams, who approach AI deployments holistically. For academia to maintain a “siloed” approach means to remain detached from best practices of the industry.

### A robust experiment requires a finance professional

We noted that almost all the academics involved in the 27 experiments we reviewed are from computer science background. We speculated that unique aspects of forecasting financial market performance (chief among them a very real and potentially devastating impact on the investors should the forecast fail) were overlooked. We believe that running multiple model configurations in parallel, and “cherry-picking” of the results were not intentionally misleading. We believe that authors did not always fully appreciate the nature of financial markets, the logistics of real-world investment process, and the impact investment decisions have on people (particularly in case of losses). We therefore strongly recommend that future experiments include a finance professional, who will ensure that experimental setup is as close to real-world conditions as possible.

### Forecasting extreme events

If Machine Learning algorithms are bound by their training data, they will not be able to forecast events more extreme than those contained in the training data. Investors have learned that over time extreme events in the financial markets tend to get even more extreme. All people can extrapolate (or at least imagine) events more extreme than those they have personally experienced or learned about—it is a basic human trait. Unless algorithms are robust and intelligent enough to do the same, their ability to forecast extreme events (and to protect the investors against them) will be substantially limited.

### Alternative data as a source of alpha

Vast majority of the 27 experiments in our review focused on elementary financial data, i.e., stock market returns and common financial statement ratios, technical indicators, or economic indicators. Only one [20] employed what we would call alternative data (Google searches, news trends, Wikipedia searches). The problem with using standard market data (especially stock market returns) is that it is very noisy, as it represents a weighted average of (often conflicting) actions and beliefs of millions of market participants. “Alternative data” is a broad term without clear boundaries: It may include satellite data of oil inventories, online shopping receipts, footfall in retail stores, or shipping traffic. Alternative data is limited by vendors’ inventiveness and clients’ willingness to pay for it. One very interesting aspect of alternative data is that it can be much narrower and more focused, and thus much more objective and much less noisy than market data. We see a promising case for using ML in combination with alternative data to predict local markets based on their unique characteristics.

### The advantages and limitations of ensemble models

Ensemble models begin to proliferate en masse from 2015 onwards, with some sporadic cases prior. It is generally accepted that ensembles outperform individual models (at least in case of ensembles of the same types of models, e.g., ensembles of neural networks or decision trees). The experiments we analyzed mostly corroborate that, although there were individual exceptions (e.g., in [21] the ensemble did *not* always outperform each individual model). Ensembles seem like a promising path to take, and one that can deliver more successful results than individual models.

While ensembles seem like the way forward (also empirically: Aidya was run by an ensemble model), the question is: what are the limits of synergy and total predictive power of an ensemble? If there were even minute marginal improvements with the addition of one more model to the ensemble, then with sufficiently high number, predictive accuracy should approach 100%. Intuitively this does not seem right. It also very closely resembles linear regression, where adding more variables can lead to “mechanical” improvement of predictive accuracy, even if some of the predictors are unrelated to the dependent variable and useless. We are not aware of this question being asked explicitly in the context of financial time series prediction, and we think it is a very interesting research area.

### A (more) plausible use case—ML in ESG

Our analysis shows that to date the AI funds are “yet to deliver Earth-shattering returns”, as Bloomberg article^4^ (presciently) put it back in 2017. However, there is one area where AI’s ability to process huge amounts of data (some structured, some not) can add tremendous value in the investment process—ESG screening. ESG stands for Environmental, Social, and Governance, and relates to broadly defined ethical and environmentally-friendly investments. Understandably, not all companies can be inherently ESG (e.g., oil companies), but even among those some will be more ESG-focused than others. For about 10 years now there has been a growing ecosystem of ESG data vendors, which, famously, differ heavily among their metrics or even ESG scores for the same entities. This is understandable: ESG is a fairly new field, the metrics are not clearly defined, and there are a lot of contradictory messages in the markets. ESG can be framed as a closed-ended problem, whose outcome will be a singular score or a set of scores across different metrics. Consequently, it sounds like a promising use case for ML, which can add a lot of value in the investment decision-making process while utilizing core strengths of ML algorithms.

### The road ahead for AI funds

The number of known cases of “pure” AI funds in the markets is not high. As mentioned before, we believe that utilization of ML in investment decision-making proper (let alone autonomous or near-autonomous investing) is still extremely low. It may not remain extremely low for much longer though. There is definitely interest in the investment industry, and willingness (among some) to try the new technology out in the markets.

China-based Zheshang Fund Management, with approx. USD 6.5bn AUM (which is a small amount in the world of investment management), planned on launching a fund investing solely on the basis of ML algorithms in Q3 2019.^5^

UK-based Baillie Gifford announced in Jan-2020 that—following over 2 years of research and development—it was considering launching a fund using AI in investment decision-making.^6^

In Nov-2019 global investment management powerhouse JP Morgan Asset Management launched a genetic therapies fund. Its portfolio managers (Yazann Romahi, Berkan Sesen and Aijaz Hussein) will employ an ML tool called ThemeBot to help identify stocks of relevance.

VT Wealth, a little-known Swiss wealth manager launched an investable ML-driven strategy in Jan-2020.

There are also start-up advisories and consultancies, which may not have the size, scale, and resources to launch their own funds, but instead want to offer their advisory services and/or products to investment managers.

Cambridge, UK-based Prowler.io promotes its versatile AI platform VUKU to investment managers as a modelling, prediction and portfolio strategy tool.

There are at least 5 other small-scale advisories and consultancies which offer stock-picking, event prediction, or market forecasting as outsourced service to existing investment managers that we came across in our research. They requested to remain anonymous.

### Looking beyond equities

Global equities market is enormous. According to World Bank, in 2018 total value of equities traded worldwide was USD 68.12 Tn [39]. However, not all markets and stocks are equally liquid, stocks are not infinitely divisible, and trading costs are non-negligible. By comparison, in the foreign exchange (FX) market, currencies are almost infinitely divisible, there is practically infinite liquidity (for the major currencies; less so for the emerging ones), and trading costs are lower as % of the transaction amount (for the major currencies). The trading volume in 2019, as surveyed by Bank of International Settlements (BIS) [40] was a staggering USD 6.6 Tn… per day. FX rates time series are quite different from equities prices time series: the former are much more stable overall and do have long-term means they revert to (e.g., the long-running historical average of 1.5 USD to 1 GBP). Again, this is more the case for major currencies; the currencies of emerging and less-developed nations may at times be very unstable (e.g., Zimbabwean dollar).

However, fx rates forecasting might overall be the most lucrative to get right due to the sheer size of the market.

### Final thought: “man versus the machine” versus “man and the machine”

As final conclusion to our article we posit that neither the Machine Learning algorithms, nor the industry, nor the regulators are yet ready for autonomous or near-autonomous investment decision-making by an AI. We are much more optimistic when it comes to AI-based investment research tools: sentiment analysis, natural language processing (NLP), earnings calls analysis, behavioural analytics, asset valuation tools, ESG screening. These skills fall into the category of “cold cognition”, in which information processing has no emotional component [41]. Those narrow-use tools can be of great help to a human decision-maker. That person will utilize their cognition (both emotional “hot cognition” and analytical “cold cognition” [42]) and their judgement, and will make the final investment decision—a decision for which they, not an algorithm, will be responsible and accountable.

## Figures and Tables

**Fig. 6:**
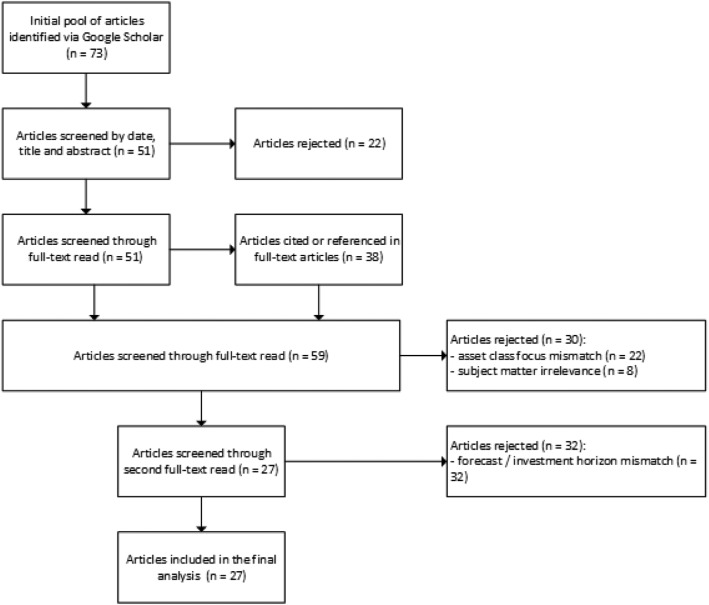
Article selection and inclusion/exclusion process

**Fig. 7:**
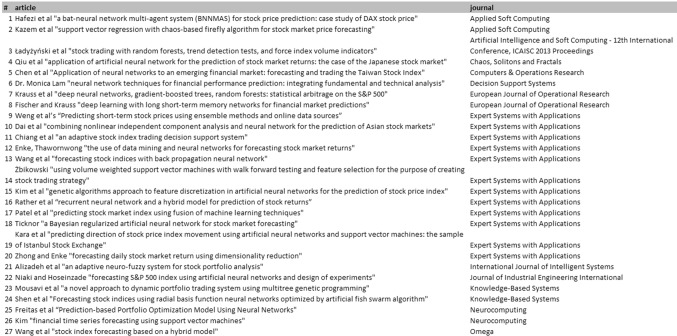
Journals breakdown

**Fig. 8:**
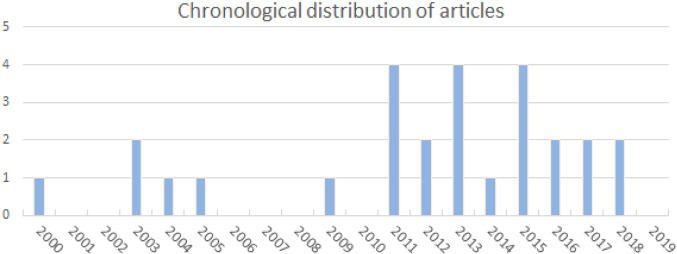
Chronological distribution of selected articles

## Appendix A: Academic articles selection criteria

Articles were eligible for inclusion in our review if they met all of the following criteria:

- They were describing empirical, first-hand experiments in AI forecasting of financial markets (proxied by market indices such as FTSE 100 or S&P 500).
- They were published in a peer-reviewed journal or conference proceedings in the past 25 years (1994–2019). Given the state of personal computing in 1994, we found it extremely unlikely that any there could be any realistic experiments prior to that date. Our initial plan was to make 2000 a cut-off point, but we were anecdotally aware of some high-impact articles dating back to the 1990′s, so we broadened the initial inclusion criteria.
- They were focused on equity (stock) market.
- They were either explicitly focused on or could be used in long-term investing (with time horizon being months/years/indefinite). We allowed articles using 1 day as basic unit of forecast, as long as the horizon of forecast was much longer.

Articles were rejected from our review if they met any of the following criteria:

- They were focused on other asset classes (fixed income, FX, private equity etc.)
- Their forecasts were focused on short time horizon (from milliseconds to hours). We do not consider such short timeframes as applicable to investment decision-making—we consider them applicable to speculation/day-trading. Day-trading is legal, and research into ultra-granular forecasts is perfectly reasonable, but it did not align with our research interests.
- They were otherwise not relevant on subject matter basis. This was the case with articles focused entirely on the computational side, with financial data being treated as a generic data set, without due consideration to its unique nature (Fig. 6).

The initial articles search was done via Google Scholar using base terms “AI market forecasting” (and its many derivatives: “AI in financial market forecasting”, “AI in stock market forecasting”, “AI in equity market forecasting”) and “AI in portfolio construction”. All terms have been tried twice: once with “AI” and once with “Artificial Intelligence”.

### Bibliographical analysis

“Expert Systems with Applications” was the most popular source by number of articles (12). Overall, 11 magazines and 1 conference paper are represented in our analysis (Fig. 7).

Majority of articles have been cited multiple times—16 articles have been cited 100 + times; only 3 articles have been cited < 20 times (Fig. 8). Mean number of citations is 228 and median is 138. Older texts have on average more citations than the more recent ones, which is understandable. Citations were an important factor in article selection, but not the critical one. High number of citations (we defined “high” as 100 +) was a very useful proxy of article’s popularity and its contribution to the field. At the same time, our primary criterion was the experiment itself, which is why we were happy to include publications with lower citation counts. We did not observe any clear relationship between experimental results (defined as forecasting accuracy) and number of citations.

## Appendix B: Glossary

- AUM—assets under management. The total value of assets within a portfolio, fund, or an investment management company.
- Banks—financial institutions in the business of (primarily) taking deposits, lending money, facilitating transfers between accounts, and accounting for given entity’s (which may be a person or a corporate) credits and debits.
- Asset managers/investment managers—financial institutions which pool their clients’ money (clients can be individuals or institutions [e.g., investing corporate pension plans’ assets]) and invest them according to clients’ preferences, risk appetite, time horizon, objective etc. The two terms are used interchangeably.
- Equity/stock—residual claim or interest of the most junior class of investors in assets, after all liabilities are paid. In simple terms, equity represents a unit of ownership of the company, usually in the form of common stock. In case of a corporate bankruptcy common stock holders are highly likely to lose all their money and not recover anything.
- Equity index/stock index—index represents a basket of equities compiled according to clearly defined criteria. The most well-known equity indices (FTSE 100, S&P 500) represent the largest listed companies in a given market, with the % weight of a given company in the index being a function of the value of its equity (market cap) as a proportion of the value of all the companies in the index.
- Bond—debt security, in which the authorized issuer owes the holders a debt and, depending on the terms of the bond, is obliged to pay interest (the coupon—if such applies) and to repay the principal at a later date, referred to as maturity.
- Return—% or monetary gain (or loss) made on an investment over a period of time.
- Risk—quantified probability of a loss, usually also including the estimated magnitude of the loss.
- Portfolio—a basket of financial instruments an individual or an institution (e.g., pension fund) invest in.
- Allocation—% breakdown of all investments within a portfolio. One portfolio can be broken down in multiple ways (e.g., by country, sector, market type, individual holdings), but in professional investments allocation is always predetermined and informs what asset universe is available to choose from. From this predetermined universe (which can be defined tightly or loosely) individual instruments are being selected into the portfolio. Alpha/active return/excess return—return above the portfolio benchmark (e.g., if the portfolio returned 11% and its benchmark returned 5% over the same period, then alpha is 6%).
- Buy and hold—investment strategy whereby the composition of the portfolio is static and does not change over time. Buy and hold is a passive strategy (no trading costs, no rebalancing, no time spent on analysis besides the initial investment decision) and is a frequent comparator for active strategies. Investing in an instrument linked to an index (e.g., an index-tracking ETF) is a particular form of buy and hold, because the investment itself is static, but the composition of the index fluctuates over time.
- Derivative—a financial instrument which does not have its own intrinsic value (defined as a claim on company assets), but whose value is derived from an underlying instrument or instruments.
- Long position—a positive holding of a financial instrument. In case of equities (and most other non-derivative instruments) long position comes with an expectation of price increase (and dividends or other periodical payments if applicable) and profit commensurate to (or above) risk taken by the investor.
- Short position—a negative holding of a financial instrument. It is somewhat counterintuitive, but it is a way of expressing (and profiting from) having a negative outlook on an instrument. Short position can be obtained “physically” by borrowing shares for a fixed period of time from someone who either owns them or keeps them in custody on behalf of the owner and then selling them in the market. After a fixed time the borrower buys back the shares in the market and returns them to the owner or the custodian. If the instrument’s price went down during that time (e.g., the short seller sold it for 90 but bought back for 60), then the short seller pockets the difference (minus a fee payable to asset owner or custodian) as their profit. The asset owner ends up with the exact same instruments they held in the beginning and some extra income from the short seller’s fee. Short exposure can also be obtained through the use of derivatives and/or through spread betting platforms (which in most cases gain exposure through the use of derivatives themselves) —both of these are most likely choices for small individual investors.

If the asset increases in price, the short seller makes a loss.

- ETF—Exchange Traded Fund. A fund which can be invested in quickly, easily, and usually cost-effectively because it trades on a stock exchange like common stock of listed companies. ETF’s are a popular way of getting exposure to equity indices.

## Footnotes

1 The investors may have negative view on certain holdings of their portfolio and at the same time have a positive view on the other, resulting in a typical long/short portfolio. An investor can also have a negative view on the entire market, and short it in a number of ways. The former is applicable to a bespoke portfolio and the latter to a market portfolio.

2 Brought to the fore at the apex of the 2008 financial crisis, when some regulators (including UK’s FSA, predecessor of FCA, temporarily prohibited short selling in a (failed) attempt to reduce market decline).

3 https://www.theguardian.com/business/2001/dec/06/personalfinancenews.unilever

4 https://www.bloomberg.com/professional/blog/machine-learning-models-often-fail-learn-quicktake-qa/

5 https://www.bloomberg.com/news/articles/2019-07-24/china-s-first-ai-fund-learned-from-the-country-s-best-traders?sref=mSzXYHhy

6 https://www.igniteseurope.com/c/2633583/318483/baillie_gifford_considers_launch_assisted_fund?referrer_module=emailMorningNews&module_order=3&code=ZDI5cVkybGxZMmd1WW5WamVubHVjMnRwUUdacGJDNWpiMjBzSURFeE1UZzBNek16TENBME56QXdORGN6TlRjPQ
